# Information-Driven Cooperative Path Planning for Multi-UAV Bearings-Only Tracking of Clustered Targets

**DOI:** 10.3390/s26216885

**Published:** 2026-10-30

**Authors:** Fuchao Xie, Jiyuan Pan, Wenguang Feng

**Affiliations:** Xi’an Research Institute of Navigation Technology, Xi’an 710068, China; pandgeon@126.com (J.P.); fengwenguang6@163.com (W.F.)

**Keywords:** multi-UAV, bearings-only tracking, clustered targets, primary–auxiliary cooperative, adaptive weighting, information-driven, RRT path planning

## Abstract

Multi-UAV bearings-only tracking of clustered targets requires coupling target state estimation with platform-motion planning. This paper proposes an information-driven cooperative path-planning method. Targets are partitioned into groups based on spatial distribution, and each UAV primarily tracks its assigned group while providing auxiliary observations to other groups when detected, forming a primary–auxiliary cooperative architecture. Intragroup path planning employs an adaptive-weighting mechanism, where target weights are computed in real time from the trace of the unscented Kalman filter predicted covariance, automatically prioritizing high-uncertainty targets in RRT-guided sampling. Intergroup collaboration adopts a distance-parameterized noise model; bearing-measurement noise is modeled as a function of observation distance, adaptively attenuating the Kalman gain of distant cross-group measurements. The convergence rate of guided sampling is shown to be proportional to the adaptive weight, and the distance-parameterized noise guarantees filtering robustness. Simulations demonstrate that, compared with fixed-weight noncooperative and cooperative planning, the proposed method reduces the RMSE by 91.2% and 15.3%, and shortens the per-step planning time by 30% and 27%, respectively, while eliminating the track-loss events observed in the baselines.

## 1. Introduction

Multiple unmanned aerial-vehicle (UAV) cooperative target tracking extends surveillance coverage and mitigates loss risks caused by target maneuvers or occlusions, and has become an important technical means in surveillance, battlefield reconnaissance, and other fields [1,2,3]. When sensors can only provide target bearing angles (bearings-only passive tracking), system observability critically depends on active platform motion; when the surveillance area expands to clustered targets (e.g., naval-vessel formations, ground-vehicle groups), the spatial aggregation and cooperative maneuvering characteristics of the targets pose special challenges to task allocation and resource scheduling: how to couple multi-target state estimation with platform-motion planning under limited platform and sensor constraints is the core problem of clustered-target bearings-only tracking.

For bearings-only tracking, existing studies have established theoretical foundations for optimal observer maneuver from an information-theoretic perspective. Logothetis et al. [4] designed optimal paths for bearings-only observers; Oshman and Davidson [5] formulated observer trajectory optimization as an optimal control problem solved under the Fisher information matrix framework; Passerieux and Van Cappel [6] derived analytical conditions for optimal maneuver in single-station bearings-only tracking. Recently, Fu et al. [7] proposed a Gaussian process-based trajectory-optimization framework for unknown maneuvering targets; Zhou and Roumeliotis [8] extended active tracking to multi-robot systems, studying cooperative target tracking fusing relative observations. Doğançay [9] analyzed the geometric factors affecting passive emitter-localization accuracy and proposed UAV path-planning strategies to improve the estimation performance. This maneuver-based mechanism reflects a universal geometric property inherent to passive sensing: in monocular vision and structure-from-motion (SfM), depth or scale unobservability is resolved through the parallax induced by camera translation [10,11]; in passive sonar and passive localization, the geometric baseline generated by observer motion is essential for establishing range observability [12,13].

For multi-platform cooperative tracking and planning, existing research has unfolded from two aspects: state estimation and platform maneuver. In state estimation, Dai et al. [14] proposed a collaborative trajectory-optimization method for asynchronous 3-D multi-target tracking under passive measurement conditions; Dames [15] achieved distributed multi-target search and tracking based on the probability hypothesis density filter and Voronoi tessellation; Anastasiou et al. [16] addressed maritime search-and-rescue scenarios by adopting a model predictive control framework that jointly optimizes UAV guidance and target state estimation, with UAV-target assignment realized through a clustering method. In platform-motion planning, Oh et al. [17] achieved coordinated standoff tracking by adjusting multi-platform spatial geometric configurations; Rao et al. [18] proposed a field-of-view sharing mechanism to alleviate tracking loss caused by occlusion through multi-platform visual information complementation. Regarding the spatial aggregation characteristics of clustered targets, Li et al. [19] proposed a resolvable group-target tracking method based on the multi-Bernoulli filter, using a virtual-leader kinematic model to characterize intra-group motion constraints; Koch [20] and Feldmann et al. [21] modeled the entire cluster as a virtual extended target with an ellipsoidal extent based on random-matrix theory, treating all measurements as independent observations that share a common centroid state and a common extent covariance matrix, thereby laying a theoretical foundation for measurement-level joint processing of group targets. In distributed fusion, the classical covariance intersection method [22] achieves conservative fusion via convex combination, but involves computationally intensive matrix inversion, which limits real-time performance and motivates the need for lightweight measurement-level collaboration.

For information-driven path planning, optimizing platform-motion trajectories to maximize information measures has become a core paradigm in robotics. Hollinger and Sukhatme [23] modeled robotic information gathering as an information-measure maximization problem under motion-budget constraints, proposing a family of sampling-based information-gathering planning algorithms with approximate performance guarantees via the submodularity of information measures; Kantaros et al. [24] extended this framework to multi-robot spatio-temporal field estimation, proposing a non-myopic sampling-planning algorithm with asymptotic optimality guarantees for long-horizon information accumulation. On UAV platforms, Popović et al. [25] proposed an informative path-planning framework for terrain monitoring combining Gaussian process environmental modeling with online replanning. Alternatively, data-driven approaches have also been explored for path planning. Bartolomei et al. [26] introduced semantic information into active perception, training deep-reinforcement-learning strategies with semantic information gain as reward; Yang et al. [27] learned continuous control policies for information-theoretic active perception; Westheider et al. [28] adopted deep reinforcement learning for multi-UAV adaptive path planning. While these data-driven methods provide flexible strategies, their reliance on extensive offline training limits their generalization in safety-critical tasks [29,30].

For online replanning under complex constraints, rapidly exploring random trees (RRTs) and their variants provide mature solution tools. Karaman and Frazzoli [31] proposed the RRT* algorithm and proved its asymptotic convergence to the global optimum with probability 1; around convergence efficiency and real-time performance, ellipsoidal heuristic sampling [32], anytime replanning [33,34], and complex-environment retraction mechanisms [35] were successively proposed; Williams et al. [36] further combined RRT with bearings-only passive tracking to achieve information-driven trajectory planning for moving targets.

In summar, conventional RRT-based planners primarily optimize geometric costs (e.g., path length and smoothness) while neglecting target estimation uncertainty [31,32,33,34,35]. Information-driven path planning has become a mainstream paradigm for target tracking [23,24,25], and to enhance efficiency for clustered targets, it has further evolved to adopt grouping strategies. However, existing grouped methods typically overlook individual tracking uncertainties within each group, leading to error accumulation for poorly observed targets [20,21]. Furthermore, data-driven approaches are often constrained by their reliance on high-quality training data, limiting their reliability in safety-critical scenarios [26,27,28,29,30]. To bridge these gaps, this paper develops an information-driven RRT path-planning method. It fundamentally reformulates the conventional RRT objective by maximizing mutual information to prioritize intra-group high-uncertainty targets, while integrating a cooperation scheme for inter-group measurement sharing, thereby ensuring primary–auxiliary reliable tracking of all individual targets while circumventing the data-dependency issues of data-driven methods. The main contributions are summarized as follows:

(1) Primary–auxiliary cooperative tracking architecture: K-means clustering is used to dynamically group clustered targets and assign them to UAVs, forming a cooperative mode of “single-UAV primary responsibility, on-demand cross-group collaboration,” balancing the global optimality of centralized allocation with the scalability of distributed execution (Section 3.1).
(2) Information-driven adaptive-weighting mechanism: the normalized value of the trace of the unscented Kalman filter (UKF) position-predicted covariance is used as the dynamic guidance weight, so that targets with higher uncertainty automatically obtain higher priority in RRT sampling, achieving adaptive tilting of observation resources toward high-uncertainty targets (Section 3.3).
(3) An on-demand communication mechanism is adopted, in which auxiliary UAVs transmit bearing measurements and their own positions to the corresponding primary tracker only when non-group targets are detected, and no communication is triggered otherwise. The distance-parameterized noise model naturally attenuates the Kalman gain of distant observations, replacing state-level fusion with measurement-level sharing and preventing distant low-quality measurements from degrading the local estimate, without requiring covariance intersection (Section 3.2).
(4) Theoretical analysis and simulation validation: the convergence rate of guided sampling is shown to be proportional to the adaptive weight, and the Kalman gain of distant cross-group observations asymptotically vanishes under the distance-parameterized noise model (Section 4). A two-phase scenario validates the method, achieving root mean square error (RMSE) reductions of 91.2% and 15.3% and per-step planning-time reductions of 30% and 27% over fixed-weight noncooperative and cooperative planning, respectively, with zero track loss (Section 5).

## 2. Problem Formulation

### 2.1. Target Motion Model

Clustered targets exhibit spatial aggregation and coordinated maneuvering, so targets within a cluster share similar motion patterns. This justifies a unified-motion model, with individual differences captured by process noise. Consider M maritime moving targets, where the state of target i at time k is

(1) $$x_{k}^{\left(i\right)}={\left[p_{k,x}^{\left(i\right)},\;\;p_{k,y}^{\left(i\right)},\;\;v_{k,x}^{\left(i\right)},\;\;v_{k,y}^{\left(i\right)}\right]}^{⊤}\in R^{4}$$

with $\left(p_{k,x}^{\left(i\right)},p_{k,y}^{\left(i\right)}\right)$ and $\left(v_{k,x}^{\left(i\right)},v_{k,y}^{\left(i\right)}\right)$ denoting the target position and velocity, respectively. A Constant Velocity (CV) motion model is adopted:

(2) $$x_{k+1}^{\left(i\right)}=F\;x_{k}^{\left(i\right)}+w_{k}^{\left(i\right)}$$

where the state transition matrix $F=\left[\begin{array}{cc} I_{2} & \Delta tI_{2}\\ 0 & I_{2} \end{array}\right]$, Δ*t* is the sampling interval, and the process noise $w_{k}^{\left(i\right)}∼N\left(0,Q^{\left(i\right)}\right)$. Its covariance follows the discrete white-noise-acceleration (DWNA) model, $Q=\sigma_{a}^{2}GG^{T},G={\left[\frac{\Delta t^{2}}{2}I_{2},\Delta tI_{2}\right]}^{T}$. *σ_a_* is the standard deviation of the equivalent white acceleration noise, and *G* maps the two-dimensional acceleration noise to the position-velocity state. The DWNA model endows the velocity channel with nonzero noise intensity, which avoids the overconfidence of a purely deterministic velocity model in bearings-only filtering.

### 2.2. Bearings-Only Measurement Model

*N* fixed-wing UAVs participate in cooperative tracking, and the position of UAV *a* at time *k* is $u_{a,k}={\left[u_{a,k,x},\;\;u_{a,k,y}\right]}^{⊤}$. Each UAV is equipped with a passive RF/ESM (Electronic Support Measures) direction-finding payload with a direction-finding sensor with maximum detection range *R*_max_. The bearings-only measurement equation for target *i* is

(3) $$z_{a,k}^{\left(i\right)}=\arctan\frac{p_{k,y}^{\left(i\right)}-u_{a,k,y}}{p_{k,x}^{\left(i\right)}-u_{a,k,x}}+v_{a,k}^{\left(i\right)}$$

where the measurement noise is $v_{a,k}^{\left(i\right)}∼N\;(0\;\;\sigma_{\theta }^{2}(∥u_{a,k}-p_{k}^{\left(i\right)}∥))$.

To capture the physical degradation of signal-to-noise ratio with distance, the bearing noise standard deviation is modeled as a function of the observation range:

(4) $$\sigma_{\theta }(∥u_{a,k}-p_{k}^{\left(i\right)}∥)=\sigma_{\theta ,0}\sqrt{1+\gamma ∥u_{a,k}-p_{k}^{\left(i\right)}{∥}^{2}}$$

where *σ_θ_*_,0_ is the baseline noise standard deviation at a reference distance and *γ* > 0 is the range-dependent attenuation coefficient. If $∥u_{a,k}-p_{k}^{\left(i\right)}∥\;>R_{\max}$, no valid measurement is obtained.

### 2.3. UAV Kinematic Constraints

Each UAV is a fixed-wing platform, subject to constraints on maximum speed *v*_max_, minimum turning radius *R*_min_, and minimum safety distance *d*_min_. The Dubins model is adopted to describe the kinematics:

(5) $${\dot{u}}_{a}=v_{\max}\left[\begin{array}{c} \cos\psi_{a}\\ \sin\psi_{a} \end{array}\right],{\dot{\psi }}_{a}=\frac{v_{\max}}{R_{\min}}\omega_{a},|\omega_{a}|\;\leq \;1$$

where *ψ_a_* is the heading angle and *ω_a_* is the normalized angular velocity control input. Any two UAVs must satisfy the collision avoidance constraint ∥*u_a_* − *u_b_*∥ ≥ *d*_min_ for *a* ≠ *b*.

### 2.4. Multi-UAV Cooperative Path-Planning Problem

At each replanning instant, given the current UAV states {*u_a_*_,*k*_} and the predicted target information $\left\{{\hat{x}}_{k+1|k}^{\left(i\right)},P_{k+1|k}^{\left(i\right)}\right\}$, the multi-UAV cooperative path-planning problem aims to determine the next position *u_a_*_,*k*+1_ for each UAV, so as to maximize the overall information acquisition efficiency. This problem can be formulated as

(6) $$\begin{array}{c} \;\underset{\left\{u_{a,k+1}\right\}}{\max}\sum_{a=1}^{N}\sum_{i=1}^{M}\alpha_{i}^{\left(k\right)}I\left(u_{a,k+1};x_{k+1}^{\left(i\right)}\;|\;Z_{k}\right)\;\\ s.t.\;\;u_{a,k+1}\in X_{\mathrm{free}},\forall a,\\ ∥u_{a,k+1}-u_{b,k+1}∥\;\geq d_{\min},\forall a\neq b,\\ \mathrm{Dubins kinematic constraints},\forall a, \end{array}$$

where *I*(⋅; ⋅∣⋅) denotes the conditional mutual information, which quantifies the information gain obtained by performing a single bearings-only observation of target *i* at position *u_a_*_,*k*+1_; $\alpha_{i}^{\left(k\right)}$ is the observation weight of target *i* (to be defined in Section 3); 𝒵*_k_* is the set of all measurements up to time *k*; and 𝒳_free_ is the feasible flight space.

Problem (6) is essentially a high-dimensional nonlinear coupled optimization problem: the objective function involves the summation of mutual information over all UAV-target pairs, and the constraints include inter-UAV collision avoidance and individual kinematic constraints. Solving this problem directly requires jointly optimizing 2*N* position variables for *N* UAVs, with computational complexity growing exponentially with the number of UAVs. Moreover, this optimization must be completed online within each replanning interval, which poses a significant challenge for onboard real-time implementation.

The subsequent sections of this paper address Problem (6) by constructing a computationally feasible decomposition framework based on target grouping, intra-group independent planning, and inter-group lightweight cooperation.

## 3. UAV Cooperative Tracking Method

### 3.1. Primary–Auxiliary Cooperative Tracking Architecture

The spatial aggregation and coordinated maneuvering of clustered targets motivate the primary–auxiliary architecture. Targets within a cluster share similar motion patterns, and are spatially proximate, enabling a single UAV to effectively observe and track its assigned group. Each UAV thus serves as the primary tracker for its group, with cross-group targets providing complementary bearing measurements when they fall within its sensing range, without explicit coordination of observation positions.

Let ${\hat{p}}_{k+1|k}^{\left(i\right)}$ denote the predicted position of target *i* at the current time. Target grouping and UAV assignment are performed by a task allocation layer (e.g., a ground station or a designated leader UAV): it collects local target estimates reported by each UAV, executes K-means clustering and Hungarian assignment, and broadcasts the grouping results {𝒢*_a_*} to all UAVs. The task allocation layer triggers regrouping and reassignment in response to the following two types of events:

(1) Periodic trigger

Regrouping is performed every *T*_cluster_ seconds to adapt to the gradual motion evolution of the target cluster. The interval *T*_cluster_ can be set equal to the replanning period Δ*t* (clustering at every step) or relaxed to a multiple of Δ*t* (e.g., every 10Δ*t*), depending on the onboard computing capacity.

(2) Platform failure trigger

When a UAV is withdrawn due to a fault, or a new platform joins the swarm, the target load must be rebalanced, and clustering and assignment are executed immediately.

Upon triggering, K-means partitions, the *M* targets into *N* groups 𝒢_1_,…,𝒢*_N_*. The cluster centers are updated iteratively as

(7) $$c_{j}=\frac{1}{|G_{j}|}\sum_{i\in G_{j}}{\hat{p}}_{k+1|k}^{\left(i\right)}$$

where the number of iterations can be capped at *K*_max_, according to the available onboard computing resources. Since the K-means objective function decreases monotonically, a local optimum is reached within a finite number of steps, satisfying real-time requirements.

Subsequently, the Hungarian algorithm assigns each group to a UAV by minimizing the total initial flight distance, with the Euclidean distance between the UAV’s current position and the group center as the cost:

(8) $$\underset{\pi }{\min}\sum_{a=1}^{N}∥u_{a,k}-c_{\pi \left(a\right)}∥$$

where *π* denotes the assignment mapping. Each UAV then becomes the primary tracker (filtering center) for its assigned target group, responsible for maintaining the state estimates and path planning for all targets within that group. The assignment is updated every several replanning intervals, to accommodate the motion evolution of the target cluster. The overall architecture is illustrated in Figure 1.

During the cooperative tracking process, each UAV actively observes the targets in its own group. In addition, targets from other groups that lie within its sensor detection range may also be observed. In this case, the UAV acts as an auxiliary tracker and transmits the cross-group bearing measurements to the corresponding primary tracker, forming a cooperative mode of “single-UAV primary responsibility, on-demand cross-group collaboration.” The task allocation layer only handles lightweight clustering and assignment coordination, while the tracking execution layer performs state estimation and path planning independently on each UAV, balancing the global optimality of centralized allocation with the scalability of distributed execution.

### 3.2. Intra-Group State Estimation and Cross-Group Cooperative Fusion

Each UAV *a* maintains an independent UKF for every target in its assigned group 𝒢*_a_*. For target *i* ∈ 𝒢*_a_*, a set of 2*n* + 1 sigma points (where *n* = 4 is the state dimension) is generated from the posterior distribution $N\left(x_{k|k}^{\left(i\right)},P_{k|k}^{\left(i\right)}\right)$. These sigma points are propagated through the CV motion model (2) and weighted to produce the predicted state $x_{k+1|k}^{\left(i\right)}$ and predicted covariance $P_{k+1|k}^{\left(i\right)}$; the standard UKF prediction equations are omitted, for brevity.

Before the measurement update, each measurement is associated by jointly exploiting the emitter identity from feature-level identification and kinematic gating [37]. Specifically, each bearing measurement is first assigned to the target indicated by the reported emitter identity, and then validated by an innovation gate (|ν| ≤ 3$\sqrt{P_{\mathrm{zz},k+1}^{\left(i\right)}}$), so that occasional mislabeled detections are rejected as outliers. Cross-group shared measurements additionally carry emitter feature labels (carrier frequency, pulse width, and PRI) and are fused only when the features match the target’s record. The predicted sigma points are propagated through the bearings-only measurement equation (3). The predicted measurement mean ${\hat{z}}_{k+1|k}^{\left(i\right)}$ and innovation covariance $P_{\mathrm{zz},k+1}^{\left(i\right)}$ are then computed, where the measurement noise variance $\sigma_{\theta }^{2}$ is calculated online via (4), according to the current UAV-to-target distance. The Kalman gain and posterior estimates are

(9) $$K_{k+1}^{\left(i\right)}=P_{\mathrm{xz},k+1}^{\left(i\right)}{\left(P_{\mathrm{zz},k+1}^{\left(i\right)}\right)}^{-1}$$

(10) $$x_{k+1|k+1}^{\left(i\right)}=x_{k+1|k}^{\left(i\right)}+K_{k+1}^{\left(i\right)}\left(z_{k+1}^{\left(i\right)}-{\hat{z}}_{k+1|k}^{\left(i\right)}\right)$$

(11) $$P_{k+1|k+1}^{\left(i\right)}=P_{k+1|k}^{\left(i\right)}-K_{k+1}^{\left(i\right)}P_{\mathrm{zz},k+1}^{\left(i\right)}{\left(K_{k+1}^{\left(i\right)}\right)}^{⊤}$$

Cross-group cooperative fusion: during observation, if UAV *a* detects a target *j* belonging to another group 𝒢*_b_* (*b* ≠ *a*), it transmits the bearing measurement $z_{a}^{\left(j\right)}$ and its own position *u_a_* to the primary tracker UAV *b*. If no cross-group targets are detected, no communication is triggered. Upon receiving the measurement, UAV *b* performs a standard UKF update (9)–(11) for target *j*, with the measurement noise variance computed via (4), using the observer distance ${\hat{d}}_{a,j}$. Since ${\hat{d}}_{a,j}$ is typically larger than the primary UAV’s distance to the target, the range-parameterized noise model (4) automatically yields a larger noise variance, and hence a smaller Kalman gain, naturally attenuating the influence of distant low-quality measurements without requiring covariance intersection or ad hoc weighting heuristics.

### 3.3. Information-Driven Adaptive-Weight RRT Path Planning

As reviewed in Section 1, unlike conventional RRT-based planners that solely optimize geometric costs (e.g., path length and smoothness) while neglecting target estimation uncertainty, and distinct from data-driven methods that require extensive offline training, the proposed information-driven RRT planner directly maximizes accumulated mutual information to prioritize high-uncertainty targets, thereby enhancing the overall tracking stability of all targets within the cluster. It builds on three components: an adaptive weight that couples each target’s priority to its filtering uncertainty (12), a mutual-information objective that evaluates the information gain of candidate observation positions (13)–(14), and a guided-sampling strategy that steers the RRT search toward the weighted targets, for fast convergence.

**Adaptive weight definition**: in this paper, the intra-group target weights are associated in real time with the filtering uncertainty. The weight of target *i* at time *k* is defined as the normalized trace of its predicted position covariance:

(12) $$\alpha_{i}^{\left(k\right)}=\frac{\mathrm{tr}\left(P_{\mathrm{pos},k+1|k}^{\left(i\right)}\right)}{\sum_{j\in G_{a}}\left(\mathrm{tr}\left(P_{\mathrm{pos},k+1|k}^{\left(j\right)}\right)\right)}$$

where $P_{\mathrm{pos}}^{\left(i\right)}$ denotes the position block (the upper-left 2 × 2 submatrix) of the state covariance matrix. A larger trace indicates higher position uncertainty, which results in a larger weight and, consequently, higher priority in the subsequent RRT path planning. This mechanism automatically directs the limited computational and motion resources toward the targets that need observation most urgently.

**Mutual information computation**: at a candidate observation position *u*, the conditional mutual information obtained by performing a single bearings-only observation of target *i* is defined as the log-ratio of the determinants of the predicted and posterior covariance matrices:

(13) $$I_{i}\left(u\right)=\frac{1}{2}\log\frac{\det\left(P_{k+1|k}^{\left(i\right)}\right)}{\det\left(P_{k+1|k+1}^{\left(i\right)}\left(u\right)\right)}$$

where $P_{k+1|k+1}^{\left(i\right)}\left(u\right)$ is the posterior covariance that would be obtained after performing a UKF measurement update with a hypothetical bearing measurement taken at position *u*, computed using the range-parameterized noise model (4) with the distance $∥u-{\hat{p}}_{k+1|k}^{\left(i\right)}∥$. When the target lies outside the detection range ($∥u-{\hat{p}}_{k+1|k}^{\left(i\right)}∥\;>R_{\max}$), no measurement would be obtained, and *I_i_*(*u*) = 0. The overall objective function is the weighted sum of mutual information:

(14) $$J\left(u\right)=\sum_{i\in G_{a}}\alpha_{i}^{\left(k\right)}I_{i}\left(u\right)$$

**RRT guided sampling**: with probability *p_g_*, guided sampling is performed. In guided sampling, a target *i* is randomly selected according to the current weight $\alpha_{i}^{\left(k\right)}$, and a guiding point is uniformly sampled on its safety distance ring $S_{i}=\left\{u|∥u-{\hat{p}}^{\left(i\right)}∥\;=d_{\min}\right\}$. With probability 1 − *p_g_*, a point is uniformly sampled in the feasible region 𝒳_free_. The RRT extends from the nearest node toward the sampled point under Dubins kinematic constraints; new nodes must satisfy the safety distance and collision avoidance constraints. The overall framework of the proposed information-driven cooperative tracking and planning method is summarized in Algorithm 1.
**Algorithm 1: Information-Driven Cooperative RRT formulti-UAV****Input**: initial target states, number of UAVs *N*, kinematic/sensor constraints1: **loop for each replanning interval** 2:  **if** clustering interval reached or UAV withdrawal/joining detected then 3:    run K-means to partition *M* targets into *N* groups via (7) 4:    run Hungarian to assign the *N* groups to *N* UAVs via (8) 5:    broadcast assignment results to all UAVs 6:  **end if** 7:  **parallel for each UAV** 8:   receive cross-group measurement (if any) 9:   perform UKF time update (prediction) for own-group targets 10:    perform UKF measurement update for own-group targets using all available measurements 11:    compute adaptive weights for own-group targets via (12) based on latest covariances 12:    run guided RRT to plan the future path: 13:     root the tree at the current UAV state; 14:     repeat until the iteration budget is exhausted: 15:      sample a target direction: with probability *p_g_*, target *i* is drawn with probability proportional to its adaptive weight $\alpha_{i}^{\left(k\right)}$; otherwise sample uniformly over the free space; 16:      extend the nearest node toward the sample subject to the kinematic constraints (*v*_max_, *R*_min_) and the safety distances (*d*_min_); 17:      evaluate the node by the path-accumulated mutual information (13)–(14); 18:    select the branch with the maximum mutual information as the planned path; 19:    move UAV along the planned path and obtain bearing measurements 20:    for each obtained measurement, attempt data association with own-group targets, if not associated, broadcast the measurement and own position 21:  **end parallel for** 22: **end loop**

## 4. Theoretical Analysis

### 4.1. Convergence Analysis of Adaptive-Weight Guided Sampling

The probability that a single guided sampling point falls within an *ϵ*-neighborhood arc segment *B_i_*(*ϵ*) on the safety distance ring of target *i* is

(15) $$P\left(\mathrm{guided point}\in B_{i}\left(\epsilon \right)\right)=p_{g}\cdot \alpha_{i}^{\left(k\right)}\cdot \frac{\mathrm{Length}\left(B_{i}\left(\epsilon \right)\right)}{\mathrm{Length}\left(S_{i}\right)}$$

where Length(*S_i_*) = 2*πd*_min_.

Let *K_i_* denote the number of sampling iterations until a tree node first enters *B_i_*(*ϵ*). Then

(16) $$E\left[K_{i}\right]=\frac{\mathrm{Length}\left(S_{i}\right)}{p_{g}\cdot \alpha_{i}^{\left(k\right)}\cdot \mathrm{Length}\left(B_{i}\left(\epsilon \right)\right)}$$

**Proof.**  The sampling iterations are independent. By (15), the hit probability in a single iteration is $p_{\mathrm{hit}}=p_{g}\alpha_{i}^{\left(k\right)}\mathrm{Length}\left(B_{i}\left(\epsilon \right)\right)/\mathrm{Length}\left(S_{i}\right)$. The random variable *K_i_* follows a geometric distribution with expectation 1/*p*_hit_, yielding (16). □

**Theorem** **1** (convergence rate and adaptive weight)**.** *For two targets i and j assigned to the same UAV, if* $\alpha_{i}^{\left(k\right)}>\alpha_{j}^{\left(k\right)}$ *and the geometric conditions are identical, then*

(17) $$\frac{E\left[K_{j}\right]}{E\left[K_{i}\right]}=\frac{\alpha_{i}^{\left(k\right)}}{\alpha_{j}^{\left(k\right)}}$$

***Physical interpretation****. When the covariance trace of target i increases, its adaptive weight *$\alpha_{i}^{\left(k\right)}$*automatically rises, and the expected number of iterations* 𝔼[*K_i_*] *decreases. Consequently, the algorithm spontaneously accelerates the observation coverage of target i. Conversely, when a target has been accurately tracked and its covariance trace shrinks,* $\alpha_{i}^{\left(k\right)}$ *decreases, and observation resources are naturally released to other targets. The convergence rate is proportional to the adaptive weight, and inversely proportional to the covariance trace*.

**Theorem** **2** (coverage guarantee of adaptive-weight guided sampling)**.** *Under the adaptive-weight guided sampling strategy:*

*(1)*  *Each target’s safety ring S_i_ is sampled with probability one as the sampling budget grows;*
*(2)*  *The neighborhood of the mutual-information maximum *$u^{*}=\arg\max_{u\in X_{\mathrm{free}}}J\left(u\right)\;$*is sampled with probability one, as the sampling budget grows.*

**Proof.**  The UKF prediction step $P_{k+1|k}^{i}=FP_{k|k}^{i}F^{⊤}+Q\;$ injects the process noise covariance *Q* at every epoch. Under the DWNA model (2), $Q=\sigma_{a}^{2}GG^{⊤}$ with *G* of full column rank and *σ_a_* > 0, so *Q* ≻ 0 and the predicted position covariance satisfies $\mathrm{tr}\left(P_{\mathrm{pos},k+1|k}^{i}\right)>0\;$ for every target. By (12), the adaptive weight is, therefore, strictly positive: $\alpha_{i}^{k}>0$.

For (i), the per-iteration hit probability given by (15) is strictly positive, since *p_g_* > 0, $\alpha_{i}^{k}>0$, and both lengths are positive. Sampling iterations are independent, so the probability of never sampling *B_i_*(*ϵ*) in *K* iterations is ${\left(1-P\left(\mathrm{guided point}\in B_{i}\left(\epsilon \right)\right)\right)}^{K}$, which tends to zero as *K* → *∞*. Because the guided point is uniformly distributed on *S_i_*, the arc segment *B_i_*(*ϵ*) can be centered at any point of *S_i_* with the same positive hit probability; hence, every point of *S_i_* is sampled with probability one.

For (ii), the maximum u^*^ of *J*(*u*) lies in the interior of some target’s detection region, since *I_i_*(*u*) = 0 outside the detection range, while *I_i_*(*u*) > 0 inside. Within this interior, *J*(*u*) is continuous, so there exists a neighborhood 𝒩(u^*^) of positive measure on which *J*, is well-defined. The uniform sampling component assigns probability

(18) $$\left(1-p_{g}\right)\cdot \frac{\mathrm{Area}\left(N\left(u^{*}\right)\right)}{\mathrm{Area}\left(X_{\mathrm{free}}\right)}>0$$

to 𝒩(u^*^). From the independence of sampling iterations, the probability that 𝒩(u^*^) is never sampled in *K* iterations decays to zero as *K* → *∞*. □

### 4.2. Filtering Robustness of Cross-Group Observations

**Theorem** **3** (distance attenuation of cross-group observations)**.** *In cross-group cooperation, when the range-parameterized noise model (4) is used and the observation distance* ${\hat{d}}_{a,j}\to \infty$, *the Kalman gain of that observation tends to zero, and the UKF update reduces to prediction only*.

**Proof.**  The UKF Kalman gain is $K=P_{\mathrm{xz}}P_{\mathrm{zz}}^{-1}$, where the innovation covariance *P_zz_* contains the measurement noise variance $\sigma_{\theta }^{2}\left(\hat{d}\right)$ From (4), as $\hat{d}\to \infty$, $\sigma_{\theta }^{2}\to \infty$, hence $P_{\mathrm{zz}}^{-1}\to 0$ and *K* → 0. The posterior state and covariance revert to their predicted values, and the observation information is completely ignored. □

**Corollary** **1** (filter consistency guarantee)**.** *Under the conditions of Theorem 3, distant cross-group observations do not increase the estimation error covariance of the primary tracking node; i.e., filter consistency is preserved.*

**Proof.**  The UKF posterior covariance is *P_k_*_+1∣*k*+1_ = (*I* − *KH*)*P_k_*_+1∣*k*_. When *K* → 0, *P_k_*_+1∣*k*+1_ → *P_k_*_+1∣*k*_; the posterior covariance does not exceed the predicted covariance, so the estimation error cannot worsen. Therefore, distant low-quality cooperative observations introduce no additional error into the filter, guaranteeing the robustness of the primary tracker’s estimates. □

## 5. Simulation Experiments

### 5.1. Experimental Setup

The simulation considers a maritime security and surveillance scenario in which a swarm of UAVs, each equipped with an RF/ESM direction-finding payload, performs bearings-only passive tracking of non-cooperative surface vessels. These vessels are equipped with onboard radar and communication systems, which continuously emit RF signals. Maritime emitter tracking is a representative application of this framework: surface vessels commonly sail in clustered formations, RF/ESM payloads provide only angular observations without range, and formation reorganization—vessels leaving one group and joining another—is a routine maneuver that stresses tracking continuity. The scenario is therefore organized into two phases that mirror these operational features. To validate the proposed method under this scenario, simulations are conducted in MATLAB R2023b on a computer equipped with an Intel Core i5–12500H @2.50 GHz processor and 16 GB RAM. The scenario and platform parameters, as well as the sensing and algorithm parameters, are listed in Table 1 and Table 2.

The scenario involves *M* = 18 moving targets organized into three subgroups (six targets per subgroup). *N* = 3 fixed-wing UAVs are deployed with initial positions, forming an equilateral triangle 3 km from the formation center. The simulation spans 1000 s, with a sampling interval of 1 s, and consists of two phases, as shown in Figure 2.

**Phase I—Aggregation (0–500 s):** the 18 targets are initially organized into three compact subgroups, each with six targets in a hexagonal formation of 1 km radius. The inter-subgroup center spacing is about 4 km, and the three subgroup centers form an equilateral triangle. All three subgroups move with a common velocity of 5 m/s along the *x*-axis, perturbed by a white acceleration noise of 0.1 m/s^2^. This phase evaluates the adaptive weight mechanism’s ability to dynamically allocate observation resources among multiple targets within each group.

**Phase II—Cross-group target transfer (500–1000 s):** at t = 500 s, two targets from each subgroup break away at 20 m/s and maneuver toward the other two subgroups. Specifically, Subgroup 1 loses two targets that maneuver toward Subgroup 2 and Subgroup 3, respectively; Subgroup 2 loses two targets toward Subgroups 1 and 3; and Subgroup 3 loses two targets toward Subgroups 1 and 2. A transferred target is regarded as having arrived when it enters the 1 km neighborhood of its destination subgroup center, after which it moves with the common formation velocity. After the transfer, each subgroup contains four original targets and two incoming targets from other subgroups. This phase evaluates (1) the responsiveness of adaptive weights to the rapid uncertainty increase in transferred targets; (2) the ability of cross-group cooperation to maintain tracking continuity for targets that leave their original primary UAV; and (3) the overall robustness of the cooperative architecture under dynamic grouping.

### 5.2. Compared Methods

Three methods are compared to isolate the contributions of adaptive weighting and cross-group cooperation. All three methods are run with identical random seeds in each Monte Carlo trial, so that they face the same target realizations and measurement noise sequences. Each method independently plans its own UAV trajectories online, so differences in observation geometry across methods reflect the planning strategies themselves.

(1) Fixed (M1)

Three UAVs, each tracking its assigned group with equal target weights (*α_i_* = 1/6). No cross-group measurements are shared.

(2) Coop-fixed (M2)

Three UAVs, each tracking its assigned group with equal target weights (*α_i_* = 1/6). Each UAV broadcasts its measurements and receives cross-group measurements from other UAVs; received measurements are fused using the range-parameterized noise model (4).

(3) Coop-adaptive (M3, proposed)

Three UAVs, each using adaptive weights computed online via (12). Each UAV broadcasts its measurements and receives cross-group measurements from other UAVs; received measurements are fused using the range-parameterized noise model (4). The full Algorithm 1 is executed on each UAV.

All methods use identical K-means clustering, Hungarian assignment, kinematic constraints, and per-step RRT replanning rooted at the current UAV state. The only differences are the weighting strategy and whether cross-group measurements are exchanged.

### 5.3. Experimental Results

#### 5.3.1. Overall Tracking Performance

Figure 3 and Table 3 compare the position RMSE of all 18 targets for the three methods, averaged over the 20 Monte Carlo runs. A run is excluded from the RMSE statistics when any target’s position error exceeds 5000 m at any time step during that run (run-level exclusion).

First, the primary–auxiliary cooperative tracking architecture dominates the accuracy gap. Fixed (M1) converges to a steady-state band of 100–250 m during the undisturbed phase (100–500 s), whereas both cooperative methods converge to 25–35 m within about 60 s and remain below 40 m thereafter. The reason is geometric: with a single observer per group, bearings-only observability depends entirely on the observer’s own lateral motion, which is limited to 50 m per step; the cooperative methods (M2 and M3) receive cross-group bearings taken from different viewing angles, which effectively triangulates the targets and removes the range ambiguity that dominates Fixed (M1)’s error. The converged-phase RMSE (650–1000 s) in Table 3 quantifies this: 484.3 m for Fixed (M1) versus 37.3 m (M2, Coop-Fixed) and 29.0 m (M3, Coop-Adaptive).

Second, all methods exhibit a temporary RMSE increase after the transfer onset at t = 500 s. For Fixed (M1) the increase is gradual but large, reaching a plateau of 550–600 m and persisting until the end of the run—the transferred targets leave the coverage of their primary UAVs, and, without auxiliary measurements, the filters cannot reconverge (two transferred targets exceed the 5000 m track-loss threshold in the process). For the cooperative methods (M2 and M3), the bump is limited to about 110 m and reconverges within about 100 s, since the transferred targets remain observed by the auxiliary UAVs during the handover.

Third, the proposed Coop-Adaptive (M3) achieves the lowest RMSE in every phase. Compared with Coop-Fixed (M2)—which shares the identical cooperative measurement set—the adaptive weighting reduces the all-time RMSE from 38.0 m to 32.2 m (a 15.3% improvement) and the converged-phase RMSE from 37.3 m to 29.0 m (22.3%). This gap is attributable solely to the weighting mechanism, since the two methods receive exactly the same measurements. Notably, Coop-Adaptive (M3) also achieves the lowest planning time. The improvements are consistent across Monte Carlo runs: the per-run standard deviations of Coop-Adaptive (±5.3 m all-time, ±7.0 m converged-phase) are the smallest among the three methods, and well below the mean gaps of Coop-Fixed, confirming that the gain is systematic, rather than incidental.

We further examine the information-gathering behavior that underlies the above accuracy differences. Figure 4 shows the per-step weighted mutual information (14)—the objective that the RRT planner maximizes—averaged over the retained runs. Coop-Adaptive maintains the highest information level throughout the run, both before the transfer and after convergence, indicating that the adaptive weights keep the UAVs at information-richer observing positions. Moreover, at the transfer onset, Coop-Adaptive exhibits a pronounced information surge, markedly exceeding the other two methods, as the adaptive weights concentrate sensing effort on the maneuvering transferred targets precisely when their uncertainty grows fastest. This transient surge coincides with the faster reconvergence reported in Table 3, and explains why Coop-Adaptive resolves the handover without track loss.

#### 5.3.2. Effect of Adaptive Weighting with Cross-Group Cooperation

The adaptive weighting (12) redistributes the observation effort within a group. Its role is best isolated by comparing Coop-Fixed (M2) and Coop-Adaptive (M3), which face identical target realizations and differ only in the weighting. Figure 5 and Table 4 shows the position RMSE of all 18 targets for the two methods, averaged over the 20 Monte Carlo runs, with lost runs excluded.

During the undisturbed phase (100–500 s), both cooperative methods maintain stable tracking performance. The position RMSE for both methods decreases rapidly after initialization, and settles within the range of 20–30 m. Notably, Coop-Adaptive (M3) consistently outperforms Coop-Fixed (M2) throughout this period, achieving an average RMSE of 20.1 m, compared to 27.3 m for Coop-Fixed (M2).

When targets maneuver to transfer between groups, the two cooperative methods exhibit characteristically distinct behaviors. In the initial transfer phase (500–550 s), the RMSE of Coop-Adaptive (M3) increases sharply, to an average of 81.9 m, exceeding the 69 m observed for Coop-Fixed (M2). This increase arises from the deliberate concentration of sensing resources by the adaptive-weighting scheme on maneuvering targets with the most rapidly growing errors, thereby temporarily reducing the priority assigned to non-maneuvering targets. This transient polarization should be interpreted as a beneficial property, rather than a limitation: because the transferred targets receive prioritized attention, Coop-Adaptive (M3) achieves reconvergence by approximately 550 s, whereas Coop-Fixed (M2) does not reconverge until about 650 s. Following convergence, Coop-Adaptive (M3) attains a lower RMSE of 30.4 m, compared with 41.0 m for Coop-Fixed (M2). More importantly, across all 20 Monte Carlo trials, Coop-Adaptive (M3) incurs zero track losses, while Coop-Fixed (M2) loses one transferred target.

#### 5.3.3. Effect of Cross-Group Cooperation on Transferred Targets

The cross-group transfer in Phase II is the most demanding part of the scenario: a transferred target leaves the coverage of its primary UAV, and must be handed over to another UAV. Figure 6 and Table 5 shows the RMSE of the six transferred targets (T5, T6, T11, T12, T17, and T18).

After the maneuver onset (500 s), Fixed’s (M1) transferred-target RMSE rises rapidly, peaks at about 830 m around 700 s, and remains above 400 m until the end of the run. Without auxiliary measurements, the primary UAV must rely solely on its own per-step motion to re-establish observability of a target that is simultaneously maneuvering away—the geometric deficit cannot be closed. In contrast, both cooperative methods limit the transfer-induced error to about 200 m and reconverge to about 50 m within 200 s, since the transferred targets are continuously observed by the auxiliary UAVs during the handover, and their measurements are fused by the new primary UAV using the range-parameterized noise model (4). The two track losses of Fixed (both transferred targets) and the single track loss of Coop-Fixed (also a transferred target) all occur in this window, confirming that the transfer is the scenario’s critical phase and that cooperation is the deciding factor.

The three lost tracks (two in Fixed, one in Coop-Fixed) share the same signature: the transferred target’s error grows linearly throughout the handover, until it crosses the 5000 m threshold, after which the run is excluded. The linear growth is the textbook signature of bearings-only observability loss—the UAV’s motion is insufficient to disambiguate the target’s range while the target simultaneously maneuvers, so the error accumulates at a near-constant rate, rather than converging. Coop-Adaptive avoids this failure mode because the adaptive weights direct the UAV toward the maneuvering targets precisely when their uncertainty grows, while the auxiliary measurements maintain a healthy covariance baseline that prevents the positive-feedback lock-in observed in single-observer configurations.

All three methods employ the same association and outlier protection in every measurement update. Each UKF update is safeguarded by NIS-based covariance inflation: the innovation ν is gated against the predicted innovation standard deviation, and a measurement is rejected when |ν| > 3$\sqrt{P_{\mathrm{zz},k+1}^{\left(i\right)}}$. A single rejection does not trigger inflation; only after three consecutive rejections for the same target is the covariance inflated by a factor ρ = 9 (followed by symmetric regularization). In addition, shared measurements are admitted only when the emitter features carried by the ESM report match the target’s feature record (Section 3.2). Since these protections are common to all three methods, the difference in track-loss count is attributable to the cooperative architecture and the adaptive weighting, rather than to the protection mechanisms. To quantify the impact of imperfect feature association, we randomly dropped each shared measurement with miss rates *ε*_miss_ ∈ {0.1, 0.2, … , 0.6} (Table 6). The proposed method maintains zero track loss at every miss rate, with the overall RMSE degrading gracefully from 32.8 m to 44.7 m—remaining an order of magnitude below the non-cooperative baseline and the best of the three methods at every miss rate.

### 5.4. Robustness Analysis

To evaluate the robustness of the proposed method under varying sensor and filter parameters, two sensitivity analyses are conducted.

#### 5.4.1. Sensitivity to Bearing Noise

The baseline bearing noise *σ_θ_*_,0_ is swept from 0.1° to 4.0°. Table 7 reports the conditional RMSE (excluding trials with track loss) and the track loss counts over 10 Monte Carlo runs. Track loss is defined as the position error exceeding 5000 m at any time step.

The results demonstrate that Coop-Adaptive maintains the lowest RMSE and zero track loss across the entire sweep range. Even at *σ_θ_*_,0_ = 4.0°, the proposed method achieves bounded RMSE (159.1 m), whereas the Fixed method suffers track loss in multiple runs. These results confirm that the effectiveness of the proposed method does not rely on a precisely tuned noise level.

#### 5.4.2. Sensitivity to Filter Process Noise

To assess the sensitivity of the proposed method to the filter process noise, *σ_a_* is swept from 0.1 m/s^2^ to 2.0 m/s^2^, while the true target maneuver noise remains fixed at 0.1 m/s^2^, and the simulation results are summarized in Table 8.

Two observations are noteworthy. First, Coop-Adaptive maintains zero track loss and stable RMSE across all noise levels, confirming that the baseline setting (*σ_a_* = 0.5) lies within a robust interval. Second, the Fixed method exhibits track loss in 4 out of 10 runs when *σ_a_* = 0.1. In contrast, the Coop-Adaptive method maintains zero track loss across the entire range, demonstrating the robustness of the cooperative architecture.

## 6. Conclusions

This paper has presented an information-driven cooperative path-planning method for multi-UAV bearings-only tracking of clustered targets, providing a training-free and theoretically guaranteed solution for reliable tracking. The method combines an intra-group adaptive-weighting mechanism, which tilts the RRT guided sampling toward high-uncertainty targets in proportion to their filtering covariance, with a lightweight cross-group collaboration strategy in which auxiliary UAVs forward bearing measurements to the responsible primary tracker on demand, fused under a range-parameterized noise model (Section 3). The convergence rate of guided sampling is shown to be proportional to the adaptive weight, and the Kalman gain of distant cross-group observations vanishes asymptotically under the range-parameterized noise model, preventing remote measurements from corrupting the local estimates (Section 4).

Monte Carlo simulations over a two-phase clustered-target scenario (Section 5) show that the proposed method reduces the RMSE by 15.3% and 91.2%, compared with fixed-weight cooperative and noncooperative planning, respectively, with a lower per-step planning time (reduced by 27% and 30%, respectively). The ablation study decomposes this improvement into two factors: cooperative measurements account for the dominant share (from 367.2 m to 38.0 m, a tenfold reduction), whereas adaptive weighting contributes a 15.3% improvement under auxiliary measurement sharing (from 38.0 m to 32.2 m) under identical measurement availability. The reliability gain is equally important: the proposed method ensures stable tracking for all targets within the cluster, dropping the track loss from 2/20 runs for Fixed to 0/20 runs. These improvements are achieved at a lower planning cost than the baselines. Simulation results validate the effectiveness of auxiliary measurements, and are consistent with the theoretical analysis.

Several directions merit further investigation. First, robust cross-group measurement association—through gating and feature identification—could be developed to further improve association accuracy. Second, the framework should be extended to heterogeneous sensor suites and communication-constrained environments. For higher-accuracy scenarios, incorporating installation-angle calibration residuals as augmented states or online-calibration variables within the filtering framework is also a worthwhile direction. Finally, validation through hardware-in-the-loop tests with actual ESM payloads and multi-UAV flight tests remains essential before practical deployment.

## Figures and Tables

**Figure 1:**
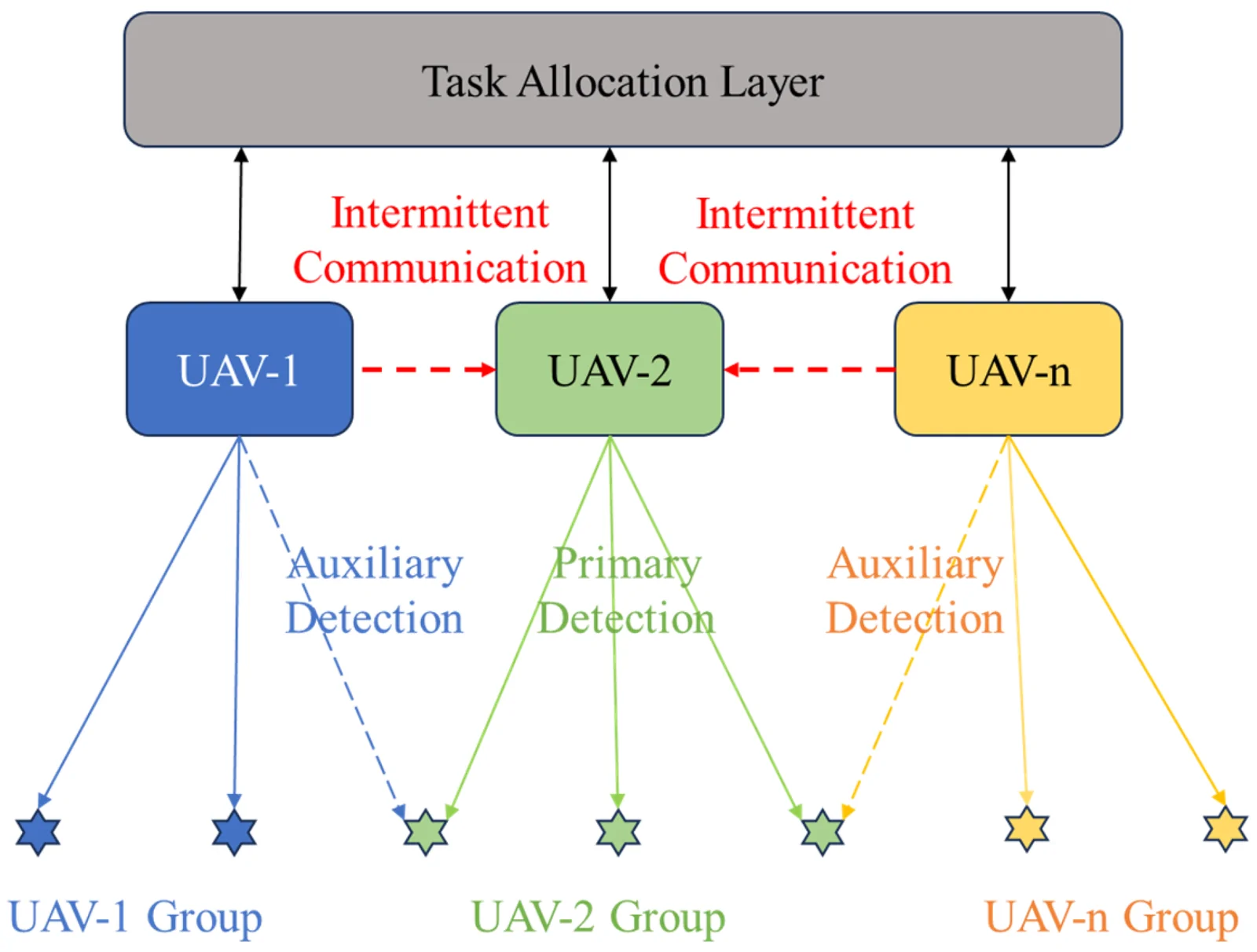
Primary–auxiliary cooperative tracking architecture.

**Figure 2:**
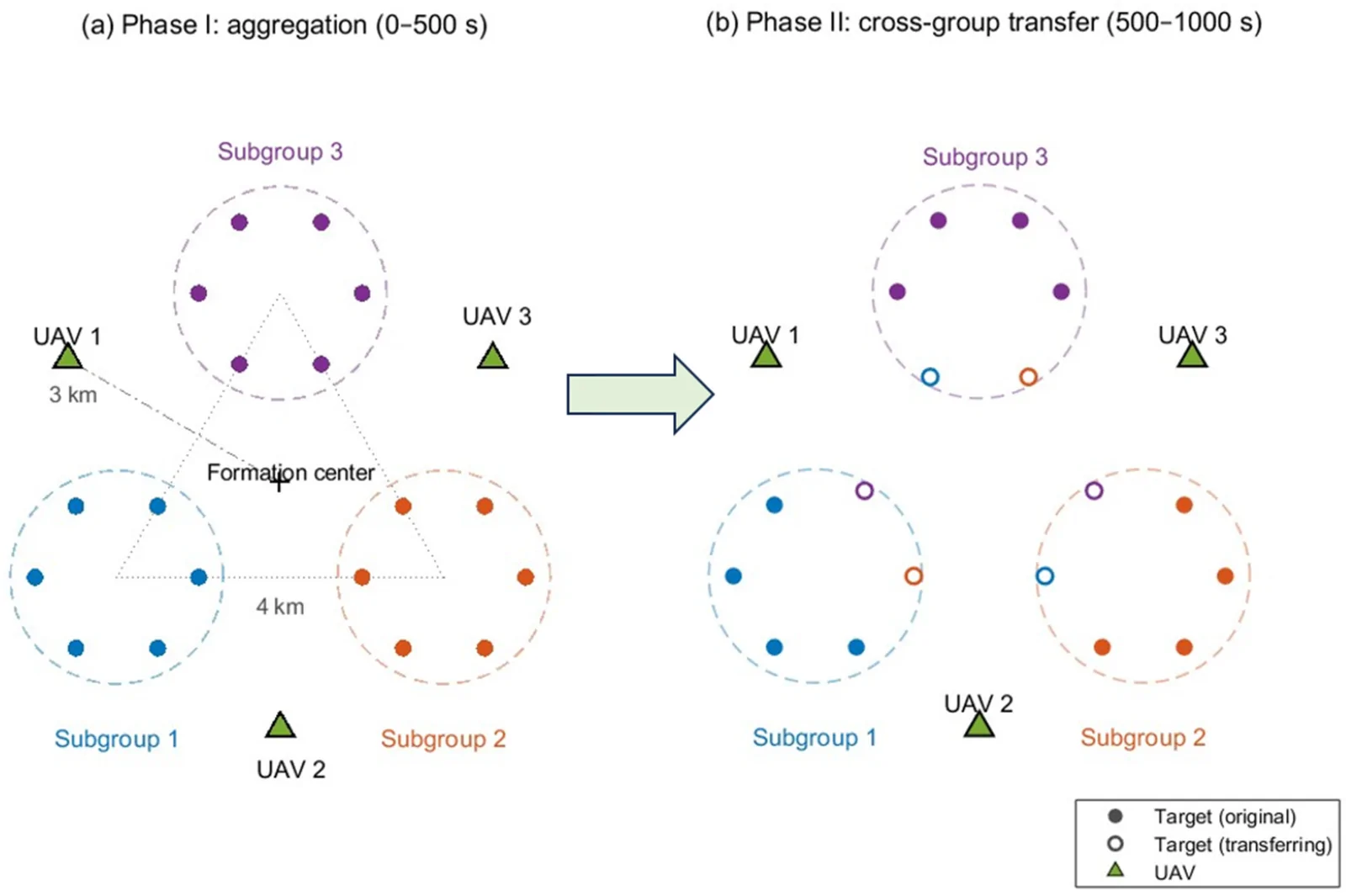
Illustration of the two-phase simulation scenario. (Blue, orange, and purple filled circles represent the original targets in Subgroups 1, 2, and 3, respectively. Hollow circles represent the transferring targets, and green triangles represent the UAVs).

**Figure 3:**
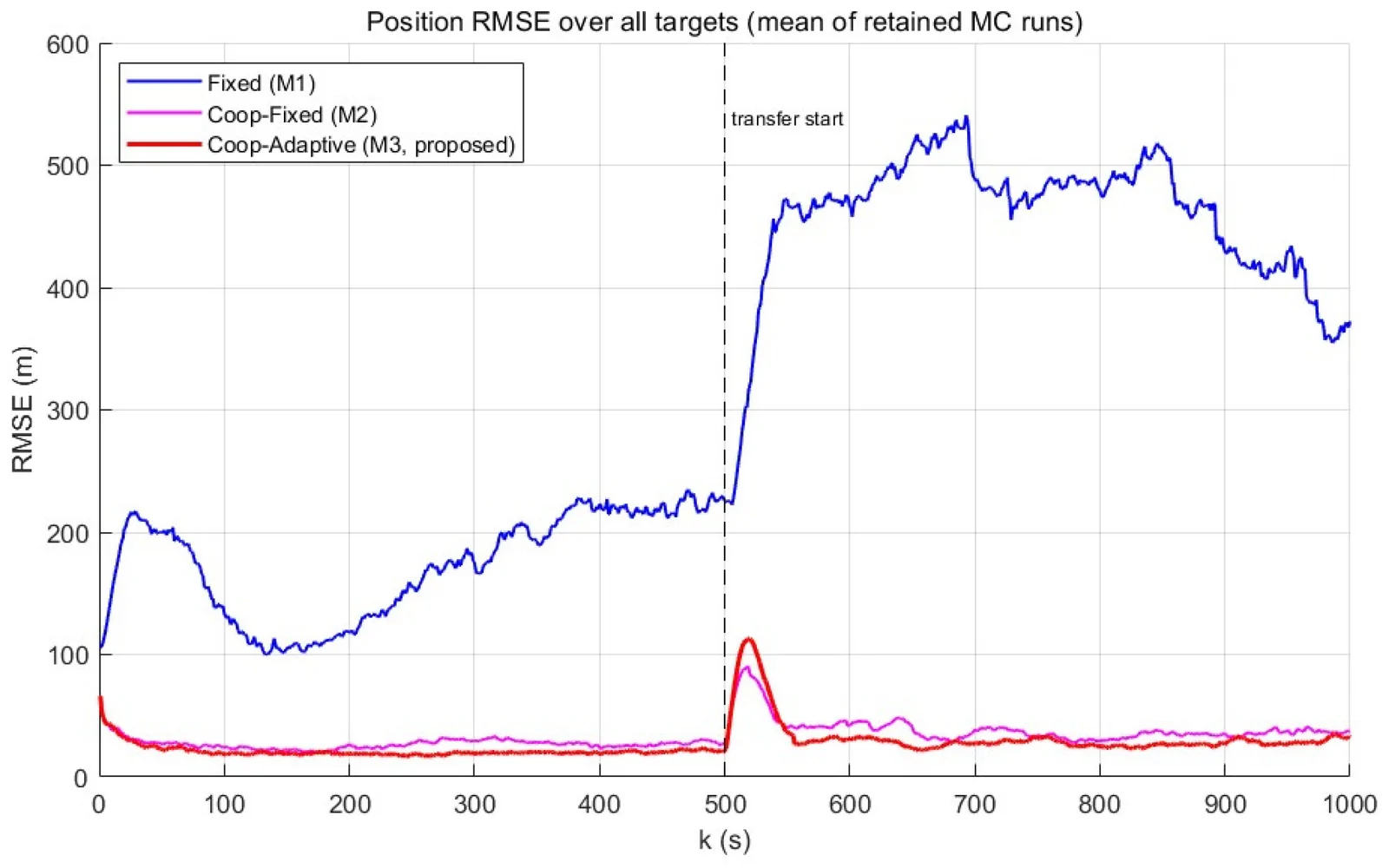
Position RMSE comparison over all targets (20 Monte Carlo runs; Fixed (M1): 18 retained runs; Coop-Fixed (M2): 19 retained runs; Coop-Adaptive (M3, proposed): 20 retained runs).

**Figure 4:**
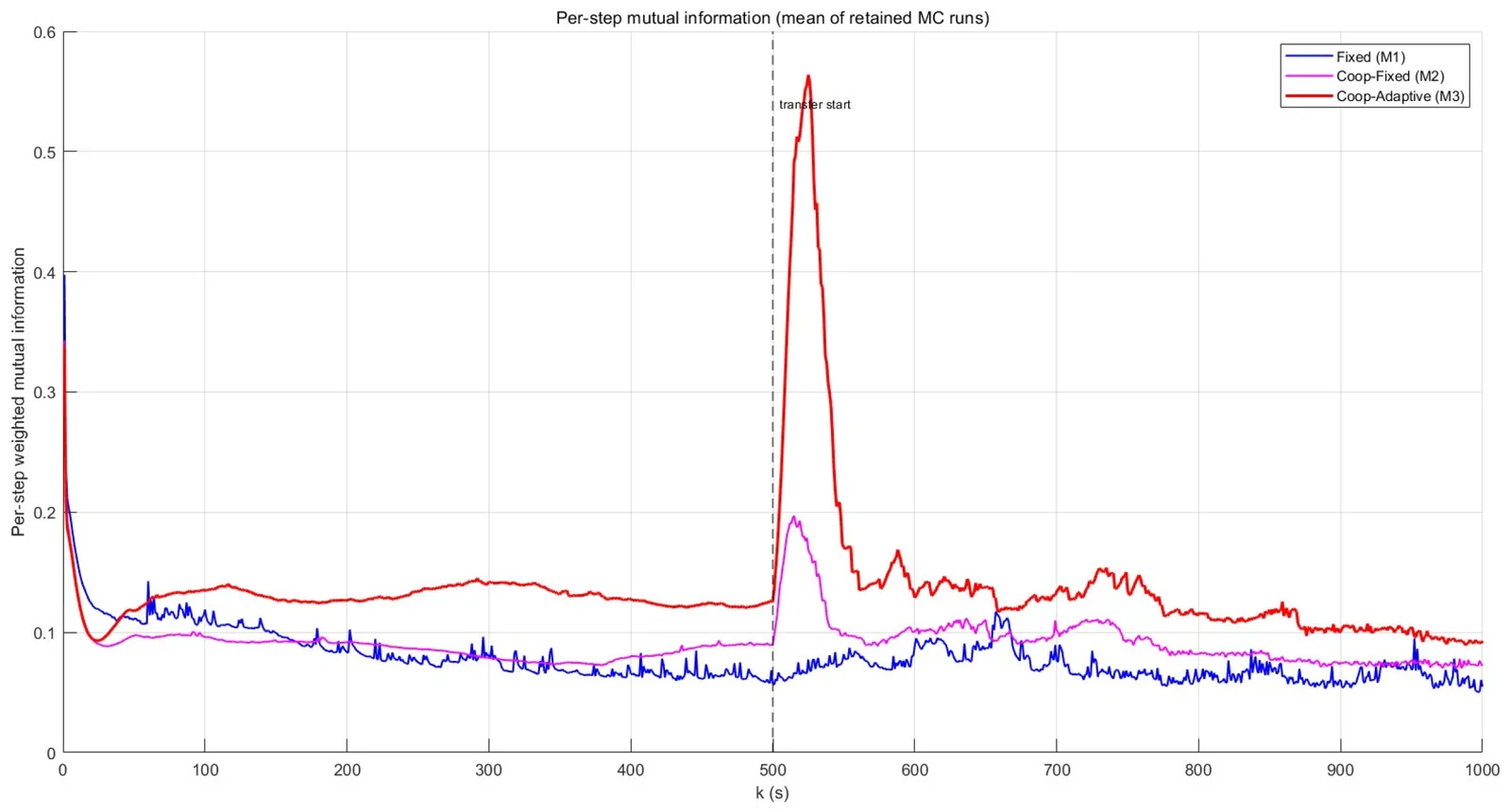
Per-step weighted mutual information (14) for the three methods, averaged over the retained Monte Carlo runs (M1: 18/20, M2: 19/20, M3: 20/20).

**Figure 5:**
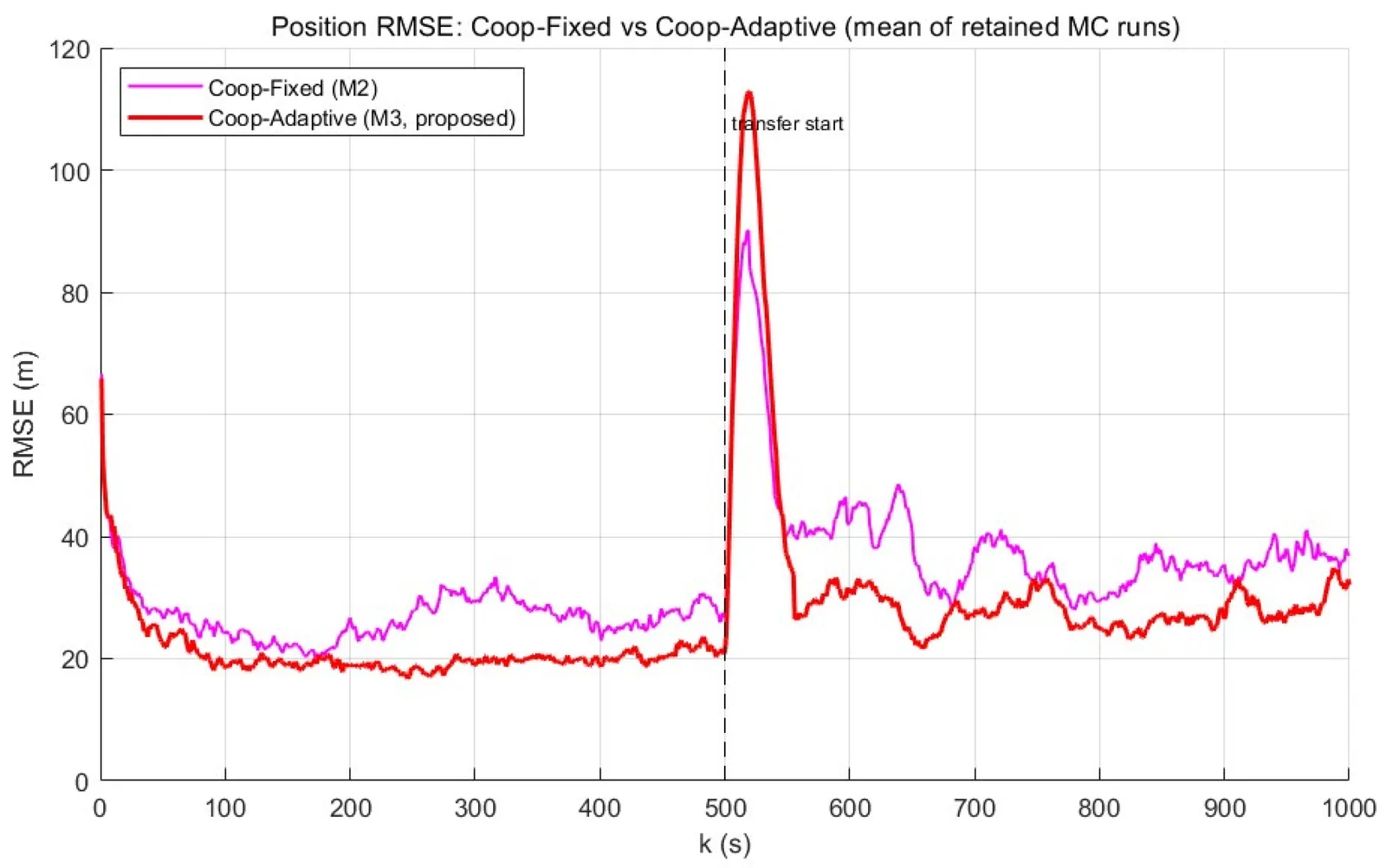
The position RMSE comparison of Coop-Fixed (M2) and Coop-Adaptive (M3).

**Figure 6:**
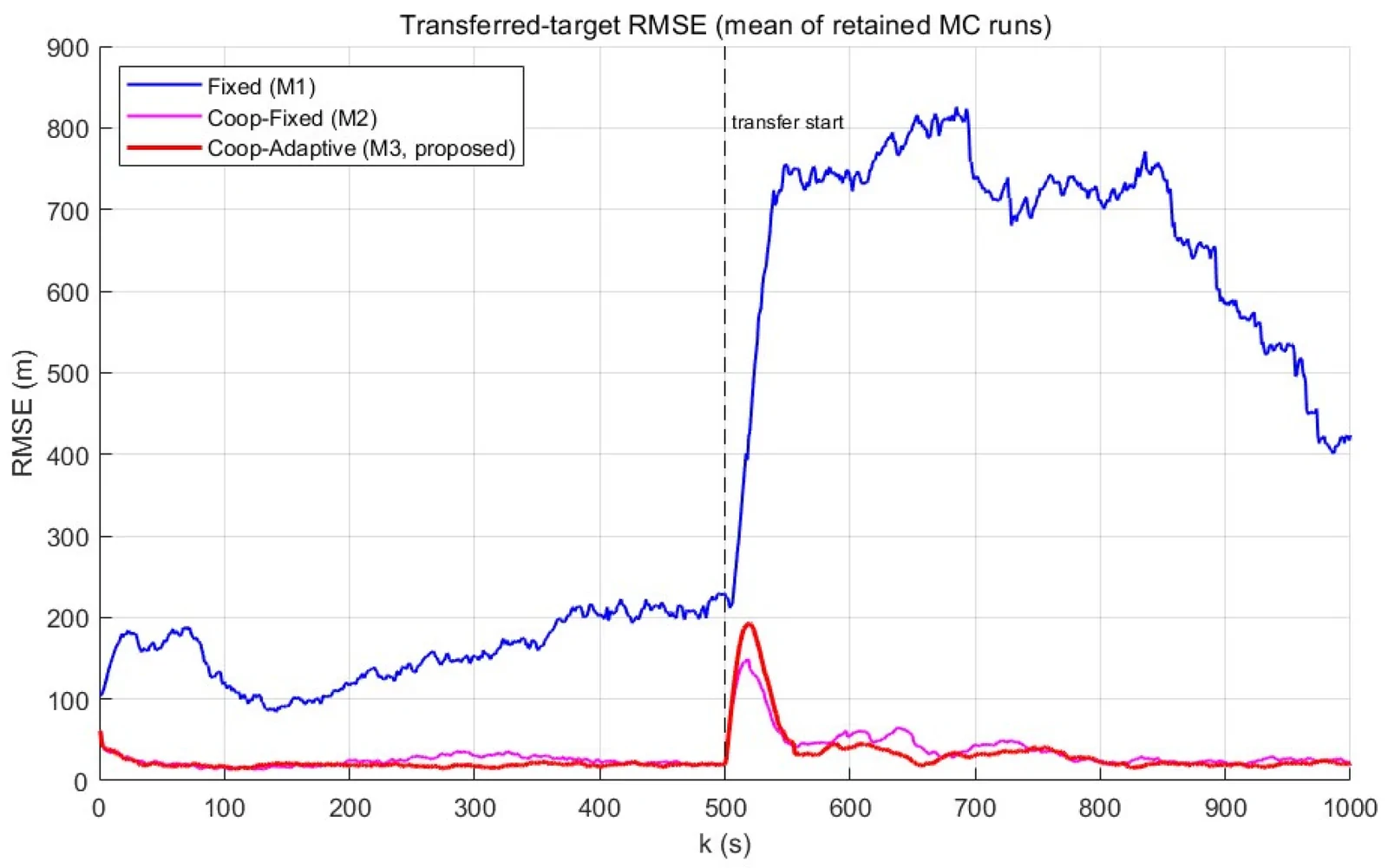
Position RMSE comparison of the six transferred targets.

**Table 1:** Scenario and platform parameters.

Parameter	Value	Parameter	Value
Number of targets *M*	18	Number of UAVs *N*	3
Subgroup formation	Hexagonal, radius 1 km	Inter-subgroup spacing	4 km
Formation velocity	5 m/s	Target maneuver noise	0.1 m/s^2^
Transfer onset time/speed	500 s/20 m/s	Transfer arrival radius	1 km
UAV initial distance from center	3 km	Maximum UAV speed *v*_max_	50 m/s
Minimum turning radius *R*_min_	200 m	Minimum safety distance *d*_min_	200 m
Simulation duration	1000 s	Sampling interval	1 s

**Table 2:** Sensing and algorithm parameters.

Parameter	Value	Parameter	Value
Filter process noise	0.5 m/s^2^	Initial position uncertainty	100 m
Baseline bearing noise std *σ_θ_*_,0_	0.2°	UKF parameters (α/κ/β)	0.1/0/2
Range attenuation coefficient *γ*	10^−6^ m^−2^	RRT guide probability *p_g_*	0.3
Maximum RRT iterations *K*_max_	1000	Maximum detection range *R*_max_	80 km
K-means max iterations	100	Regrouping interval	10 s

**Table 3:** Performance comparison (mean ± SD, 20 Monte Carlo runs).

Method	RMSE, Retained Runs (m)			Track Loss	RMSE, All Runs (m)	Planning Time (s)
	All Time	500–650 s	650–1000 s			
Fixed (M1)	367.2 ± 133.2	450.9 ± 150.5	484.3 ± 229.6	2/20	380.8 ± 133.4	0.0505
Coop-Fixed (M2)	38.0 ± 7.9	56.8 ± 26.4	37.3 ± 8.9	1/20	179.9 ± 634.7	0.0483
Coop-Adaptive (M3)	32.2 ± 5.3	56.1 ± 15.1	29.0 ± 7.0	0/20	32.2 ± 5.3	0.0355

**Table 4:** RMSE comparison of Coop-Fixed and Coop-Adaptive (mean ± SD across retained runs).

Method	RMSE 100–500 s (m)	RMSE 500–550 s (m)	RMSE 550–1000 s (m)
Coop-Fixed (M2)	27.3 ± 1.8	69.0 ± 30.0	41.0 ± 11.7
Coop-Adaptive (M3)	20.1 ± 1.6	81.9 ± 30.4	30.4 ± 6.4

**Table 5:** RMSE of the six transferred targets (mean ± SD across retained runs).

Method	RMSE 100–500 s (m)	RMSE 100–550 s (m)	RMSE 550–1000 s (m)
Fixed (M1)	175.1 ± 59.0	543.7 ± 129.9	734.3 ± 366.2
Coop-Fixed (M2)	26.0 ± 3.1	110.3 ± 55.5	47.2 ± 24.5
Coop-Adaptive (M3)	20.2 ± 1.8	137.4 ± 54.3	35.6 ± 8.7

**Table 6:** RMSE under cross-group measurement miss rates (RMSE across retained runs: mean ± SD; track loss: lost/total).

Method	0.1	0.2	0.3	0.4	0.5	0.6
Fixed (M1)	367.2 ± 133.2 (2/20)					
Coop-Fixed (M2)	41.6 ± 8.0 (0/20)	43.8 ± 7.5 (1/20)	44.1 ± 5.4 (0/20)	46.8 ± 9.2 (0/20)	48.7 ± 10.1 (0/20)	53.1 ± 11.0 (1/20)
Coop-Adaptive (M3)	32.8 ± 3.6 (0/20)	34.8 ± 6.2 (0/20)	35.1 ± 4.4 (0/20)	39.4 ± 5.9 (0/20)	41.3 ± 6.7 (0/20)	44.7 ± 8.4 (0/20)

**Table 7:** Tracking performance under bearing noise sweep (RMSE across retained runs: mean ± SD; track loss: lost/total).

Method	0.1°	0.2°	1.0°	2.0°	4.0°
Fixed (M1)	332.9 ± 130.9 (2/10)	381.4 ± 119.0 (0/10)	438.8 ± 63.5 (0/10)	452.6 ± 75.8 (3/10)	481.8 ± 42.6 (0/10)
Coop-Fixed (M2)	23.9 ± 4.3 (0/10)	35.9 ± 5.2 (0/10)	107.2 ± 14.9 (0/10)	158.2 ± 13.9 (1/10)	240.4 ± 24.0 (0/10)
Coop-Adaptive (M3)	18.7 ± 2.1 (0/10)	29.2 ± 2.9 (0/10)	76.7 ± 10.0 (0/10)	108.7 ± 9.5 (0/10)	159.1 ± 12.8 (0/10)

**Table 8:** Tracking performance under filter process noise sweep (RMSE across retained runs: mean ± SD; track loss: lost/total).

Method	0.1	0.3	0.5	1.0	2.0
Fixed (M1)	569.8 ± 104.1 (4/10)	390.8 ± 123.5 (0/10)	381.4 ± 119.0 (0/10)	452.1 ± 176.9 (0/10)	643.7 ± 135.4 (0/10)
Coop-Fixed (M2)	100.3 ± 33.9 (0/10)	43.0 ± 10.7 (0/10)	35.9 ± 5.2 (0/10)	42.8 ± 17.7 (0/10)	43.4 ± 5.4 (0/10)
Coop-Adaptive (M3)	72.8 ± 13.0 (0/10)	35.9 ± 5.6 (0/10)	29.2 ± 2.9 (0/10)	29.6 ± 2.3 (0/10)	34.8 ± 1.6 (0/10)

## Data Availability

The simulation data generated and analyzed during the current study are available from the corresponding author upon reasonable request.
